# Authors’ response: Estimating the generation interval for COVID-19 based on symptom onset data

**DOI:** 10.2807/1560-7917.ES.2020.25.29.2001269

**Published:** 2020-07-23

**Authors:** Cécile Kremer, Tapiwa Ganyani, Dongxuan Chen, Andrea Torneri, Christel Faes, Jacco Wallinga, Niel Hens

**Affiliations:** 1 I-BioStat, Data Science Institute, Hasselt University, Hasselt, Belgium; 2 Centre for Infectious Disease Control, National Institute for Public Health and the Environment, Bilthoven, the Netherlands; 3 Leiden University Medical Center, Leiden, the Netherlands; 4 Centre for Health Economics Research and Modelling Infectious Diseases (CHERMID), Vaccine and Infectious Disease Institute, University of Antwerp, Antwerp, Belgium

**Keywords:** generation interval, serial interval, COVID-19, likelihood, assumptions

To the editor: We are grateful for the comments provided by S. Bacallado, Q. Zhao and N. Ju [1]. With this reply we wish to clarify the concerns that were raised and provide some more insights.

## Assumption of independence between incubation period and generation time

By expressing the density of the serial interval Z_i_ as a convolution of X_i_ and Y_i_, we indeed make the simplifying assumption that the incubation period of the infector, v_(i)_, is independent of the generation time *X_i_* = *t_i_* − *t_v(i)_*. The possible correlation between the incubation period of the infector and the generation time should be taken into account. Ideally, both should be estimated from the same data. Unfortunately, we did not have these data directly available. Instead we assumed these quantities to be independent and we acknowledge that this assumption may not be realistic. However, to the best of our knowledge, the literature does not yet report clear indications of a strong relation between infectiousness and incubation period for coronavirus disease (COVID-19), with highly varying findings between studies [1]. It should be kept in mind that if our assumption of independence is not valid, our model is mis-specified as the convolution *Z* = *X* + *Y* is defined for independent random variables.

Liu et al. [3] have investigated the impact of correlation between incubation period and serial interval on estimates of presymptomatic transmission. They found that, in the presence of active case finding and assuming a mean serial interval of 4.8 days and mean incubation period of 5.2 days, the percentage of presymptomatic transmission was 48% when assuming no correlation, ranging from 38% under positive correlation to 47% under negative correlation. Our estimate of the proportion of presymptomatic transmission in Singapore (i.e. 48%; where the mean serial interval was 5.21 days when allowing only positive serial intervals and assuming a mean incubation period of 5.2 days) is in line with these estimates, with the credible interval (32–67%) also covering the lower estimate.

## Assumption of independent serial intervals

Our likelihood function

$$L\left(\Theta |z_{i},\;v\left(i\right)\right)\;=\;\prod_{i=2}^{n}\frac{1}{J}\sum_{j=1}^{J}f\left(z_{i}-y_{j}|\Theta \right)$$

is indeed an approximation assuming that the serial intervals Z_i_ are independent and identically distributed. Although our estimates are in line with those from other studies [4], we cannot be completely sure how much the assumption of independent serial intervals affects our estimates since those studies might involve the same or other statistical issues as well. This simplifying assumption of independent serial intervals is commonly used (e.g. [5]). An advantage of our method of inferring serial and generation intervals is that we take into account the infectious history of individuals by inferring these quantities based on an epidemic tree. This is in contrast to other studies that have estimated serial intervals using only probable infector–infectee pairs from different settings (e.g. [6]), which could lead to bias by not accounting for infectious histories.

It would be interesting to investigate the effect of this dependency between serial intervals in future work. These may be especially important when considering superspreading events, where there can be long transmission chains of the type *i* → *j*, *i* → *k*, *i* → *l*, *i* → *m*, …, when the same individual *i* generates a large number of secondary cases.

## Possibility of cycles in the Singapore data

We have indeed overlooked the fact that the way contacts are defined in the Singapore data may lead to cycles in the network when allowing serial intervals to be negative, when not accounting for directionality (i.e. a case cannot infect any of its ancestors). This could potentially lead to biased estimates of serial and generation intervals. When only allowing missing serial intervals to be positive, the problem of cycles in the network does not occur since infector–infectee pairs are then based on dates of symptom onset.

We have re-analysed the Singapore data to investigate whether this would substantially change our results. In the original Markov chain Monte Carlo algorithm, we implemented a condition that the sampled network will only be accepted if it does not contain cycles. For computational reasons we make the comparison for a scenario where we allow serial intervals to be negative up to −3 days and sample a network every 100th iteration. Table 1 shows our estimates of the generation and serial interval as well as the proportion of presymptomatic transmission *p*. We can see that the estimates of the serial/generation interval when removing cycles are a bit higher, but the credible intervals largely overlap with those of the original analysis. As expected, the proportion of pre-symptomatic transmission is a bit lower now, but again with overlapping credible intervals. Hence, we do believe our overall conclusions in the original article would not change but for future work, one should make sure that no cycles in the network occur. We have also added an update of the R code on GitHub [7].

**Table 1:** Comparison of estimates of key epidemiological parameters based on analysis with and without cycles in the infection network, COVID-19 pandemic, Singapore, 21 January–26 February 2020

Analysis	Interval	Mean		Standard deviation		*p*	
		Estimate	95% CrI	Estimate	95% CrI	Estimate	95% CrI
With cycles	GI	4.51	2.49 to 6.58	3.39	1.06 to 6.92	0.60	0.41 to 0.82
	SI	4.50	−4.75 to 15.63	5.21	4.10 to 7.78	NA	
Without cycles	GI	4.94	3.31 to 6.83	3.09	1.01 to 6.11	0.55	0.37 to 0.73
	SI	4.95	−4.21 to 15.49	5.03	4.09 to 7.28	NA	

CrI: credible interval; GI: generation interval; NA: not applicable; SI: serial interval; p: proportion pre-symptomatic transmission.

The estimates are based on an analysis allowing for negative serial intervals up to −3 days and used an incubation period with mean 5.2 and standard deviation 2.8 days.
